# Is There an Association between Food Consumption According to the Degree of Processing and Body Image (Dis)satisfaction in Adolescents?

**DOI:** 10.3390/nu15092102

**Published:** 2023-04-27

**Authors:** Juliana Ramos Carneiro, Susana Cararo Confortin, Poliana Cristina de Almeida Fonseca Viola, Antônio Augusto Moura da Silva

**Affiliations:** 1 Department of Public Health, School of Medicine, Federal University of Maranhão, São Luís 65020-905, MA, Brazil; juliana.ramoscarneiro@hotmail.com; 2 Department of Public Health, Postgraduation Program in Collective Health, Federal University of Maranhão, São Luís 65020-905, MA, Brazil; 3 Health Sciences Center, Nutrition Department, Federal University of Piauí, Teresina 64049-550, PI, Brazil

**Keywords:** food consumption, ultra-processed foods, body image, body dissatisfaction, adolescents

## Abstract

(1) Background: Adolescence is characterized by changes in eating habits, with increased consumption of ultra-processed foods and reduced intake of unprocessed or minimally processed foods, which can affect body image satisfaction. Thus, this study aims to verify the association of food consumption according to the degree of processing with body image (dis)satisfaction in adolescents from the 1997/1998 birth cohort of São Luís, Maranhão. (2) Methods: A cross-sectional study was conducted with 2515 adolescents aged between 18 and 19. Food consumption was stratified based on the NOVA classification in culinary preparations, processed foods, and ultra-processed foods, categorized into tertiles. Body image (dis)satisfaction was evaluated based on the Stunkard Body Scale and was classified as satisfaction, dissatisfaction with thinness, and dissatisfaction with excess weight. Multinomial logistic regression was used for associations. (3) Results: Among the adolescents, 77% considered themselves dissatisfied with their body image, with 42.8% dissatisfied with being thin and 34.2% dissatisfied with excess weight. However, food consumption, according to the degree of processing, was not associated with body image (dis)satisfaction. (4) Conclusion: This work highlighted the prevalence of body image dissatisfaction among adolescents but found no association between body (dis)satisfaction and food consumption according to the degree of processing.

## 1. Introduction

Adolescence involves several physical changes, biological, psychological, cognitive, emotional, and behavioral characteristics of the period known as puberty [1]. Among other aspects, changes in physical appearance can affect young people’s body image and satisfaction [2]. 

Body image comprises the mental conception of the real body [3]. In Brazil, data from the National Survey of School Health–PeNSE (2015) showed that 84.1% of adolescents considered body self-image an important or very important item [4]. Concerns about body image in young people can be influenced by several conditions, such as biological factors (age, gender [5,6,7], body mass index—BMI [8]), psychological aspects (depression and low self-esteem) [9], social factors (pressure from parents, friends, and the media) [5,7,9], or behavioral aspects (such as eating patterns) [10,11]. Due to increased independence in adolescence, young people begin making their own food choices, often subjecting themselves to diets of lower nutritional quality [12,13]. Studies conducted among adolescents have highlighted a possible relationship between distortion of body perception or body dissatisfaction and unhealthy eating habits [8,14,15,16], such as higher consumption of fast food [17] and other foods with high energy density [11]. Adopting unhealthy eating behaviors can lead to unwanted weight gain, contributing to the development of eating disorders, anxiety, depression, and increased dissatisfaction with body image [5,15].

Food processing can be considered one of the main determinants of food quality and health [18,19]. In this sense, the NOVA food classification was developed based on the degree and objective of industrial food processing. This classification categorizes foods into unprocessed or minimally processed foods, processed culinary ingredients, processed foods, and ultra-processed foods [20,21]. Unprocessed and minimally processed foods (such as vegetables, fruits, nuts, and fish) are considered markers of healthy eating due to their nutrient richness and antioxidant and anti-inflammatory properties [22]. Studies also show that higher consumption of this food group is related to satisfaction with body image [23,24]. 

Ultra-processed foods (such as fast food and sugary drinks, among other industrialized foods) are markers of an unhealthy diet due to their nutritional imbalance and the damage they can cause to health [25]. The consumption of this food group is identified as a risk factor for non-communicable chronic diseases [25,26] and increased obesity in adolescents and adults [27,28]. Thus, excessive consumption of ultra-processed foods leads to an anthropometric pattern that contradicts the thin figure idealized by society and imposed by the media, resulting in increased body dissatisfaction [29]. However, evidence also suggests an association between food consumption and low satisfaction with appearance regardless of BMI [30].

On the other hand, the inverse association is also possible, in which the perception of body image is a determining factor in eating behavior [11,31]. From this perspective, studies conducted with adolescents and young adults indicate that dissatisfaction with thinness, i.e., the desire to gain weight, leads to greater consumption of processed foods [32,33,34] and lower consumption of fruits [32]. Conversely, dissatisfaction with excess weight, which implies the desire to lose weight, leads to lower consumption of unhealthy foods with high energy density [30], such as ultra-processed foods. 

To date, few studies [23,35] have assessed the relationship between adolescents’ body image and food consumption according to the degree of food processing. Hence, this study aims to verify the association between food consumption, according to the degree of food processing, and body image (dis)satisfaction in adolescents from the 1997/1998 birth cohort of São Luís, Maranhão. We hypothesize that higher consumption of ultra-processed foods and lower consumption of unprocessed or minimally processed foods are associated with greater dissatisfaction with body image in adolescents.

## 2. Materials and Methods

### 2.1. Study Population and Data Collection

This cross-sectional study originated from a birth cohort conducted with adolescents born in São Luís, state of Maranhão, Brazil, in 1997/1998. This study uses data from the third phase of the 1997/1998 São Luís Birth Cohort [36,37]. 

The first phase of this cohort used one in seven deliveries performed between March 1997 and February 1998 as a sample basis, in ten public and private hospitals in the city, with proportional sharing according to the number of births in each unit, totaling a sample of 2493 live births. The cohort was followed up in 2005/2006, with a participation rate of 72.7%, totaling 673 children aged 7 to 9 years. Another follow-up was carried out in 2016 with 684 adolescents aged between 18 and 19 years. However, the decision to open the cohort was made to increase the size of this sample due to the small number of individuals who agreed to participate in the study in previous phases, in addition to preventing future losses and increasing the sampling power. Thus, new participants born in São Luís in 1997, who were not initially part of the cohort, were drawn. These individuals were identified using the Brazilian Live Birth Information System, school or university registration, and social network, recruiting 1828 new adolescents, which were included in the study along with the initial sample, totaling 2515 participants in the final sample [36]. The new participants went through the same steps and answered the same questionnaires as the adolescents in the original cohort. Additionally, their perinatal data were obtained retrospectively, with a questionnaire answered by the mothers.

Data collection was carried out between January and November 2016 and consisted of individual interviews and body composition assessments.

### 2.2. Dependent Variable

The assessment of (dis)satisfaction with body image was performed using the Body Scale by Stunkard, et al., [38]. This scale contains a sequence of nine body images for each gender, numbered from 1 to 9. Among these, the participants chose two figures: one with which they identified themselves or considered their current silhouette (Real Body Image—RBI) and another image that represented the body they wanted to have or considered ideal (Ideal Body Image—IBI).

Thus, body (dis)satisfaction was evaluated based on the subtraction of the IBI number from the RBI number, with the result ranging from minus eight to eight. Participants whose subtraction result was equal to zero were considered satisfied, i.e., those whose RBI was equal to the IBI. Negative results (from minus eight to minus one) were considered dissatisfied due to thinness, and positive results (from one to eight) were considered dissatisfied due to excess weight [39]. This classification does not consider BMI values.

### 2.3. Independent Variables

Food consumption was assessed using the validated food frequency questionnaire (FFQ) [40]. The 24 h food recall was the reference instrument used to validate the FFQ [41]. The FFQ contained 106 foods or food groups; eight consumption frequency options (never or <1 time/month; 1 to 3 times/month; 1 time/week; 2 to 4 times/week; 5 to 6 times/week; 1 time/day; 2 to 4 times/day; ≥5 times/day); and the size of the average portion of reference. Thus, individuals could report their consumption frequency and estimate if their portion usually consumed was smaller, equal, or larger than the one presented. Based on a reference table, the portion size was converted into grams or milliliters [42]. Nutritionists and scholars applied the questionnaire with a photo album to help estimate the portions consumed.

The reported consumption frequency was converted to daily frequency and then associated with serving size to estimate each food item’s total energy and daily energy consumed. The nutritional composition of the diet was obtained based on the nutritional composition table of foods consumed in Brazil, which is based on food composition tables and includes information from food labels and regional recipes [43,44,45].

Although the NOVA classification divides foods into four groups, this study categorized the FFQ items into three groups: 1. culinary preparations (including unprocessed or minimally processed foods and culinary ingredients) [46], 2. processed, and 3. ultra-processed foods. Culinary ingredients consist of substances, such as sugar, salt, and oils, used to season and prepare unprocessed or minimally processed foods. Since these two groups are usually combined in culinary preparations, this study classified them into a single group [19,46]. The percentage energy contributions of the culinary preparations, processed, and ultra-processed foods were estimated concerning the Total Energy Value (TEV) of the diet. The percentages of food groups were categorized into tertiles—Culinary preparations (1st tertile < 52.5%; 2nd tertile from ≥52.5% to <63.7%; and 3rd tertile ≥ 63.7%); processed foods (1st tertile < 2.8%; 2nd tertile from ≥2.8% to <5.3%; and 3rd tertile ≥ 5.3%), and ultra-processed foods (1st tertile < 27.7%; 2nd tertile from ≥7.7% to <39.1%; and 3rd tertile ≥ 39.1%). Thus, the percentage of energy from each tertile of the food groups was obtained.

### 2.4. Complementary Variables

Based on the literature [14,24,34], the following variables were evaluated as confounding factors: gender (female and male), age (18 and 19 years), self-reported skin color (white, black, brown; indigenous people and Asians were not included due to the small sample size), years of schooling (0 to 8 years, 9 to 11 years, 12 years or more), economic class according to the criteria of the Brazilian Association of Research Companies—ABEP (A, B, C, D–E) [47], and family income in minimum wages (≤3 minimum wages, 3.1 to 6 minimum wages, >6 minimum wages)—the minimum wage was equivalent to BRL 880.00 in 2016, and to BRL 937.00 in 2017. Leisure time physical activity (yes or no) was assessed using the Self-Administered Physical Activity Checklist (SAPAC) [48]. This instrument assesses the level of weekly physical activity based on the frequency, duration, and intensity of reported physical exercises. The intensity is determined by the Metabolic Equivalent of Tasks (MET), which corresponds to the energy expenditure of the activity. Thus, expenditures of 3 to 5.9 METS were classified as moderate physical activities, and those with MET ≥ 6.0 were classified as intense activities [49]. The level of physical activity recommended by the WHO is at least 150 min of moderate-intensity or 75 min of vigorous-intensity (aerobic physical activity) per week [50]. Thus, adolescents who complied with this recommendation were categorized as “Yes”, and those who did not were classified as “No” in the leisure time physical activity variable. Screen time [51] (≤2 h a day, >2 h and ≤5 h a day, >5 h a day) was considered the exposure to electronic devices such as cell phones, tablets, computers, videogames, and televisions in hours during weekdays; weekends were not considered in the evaluation. Current consumption of alcoholic beverages (no or yes), by the Alcohol Use Disorder Identification Test (AUDIT) [52], and current smoking (no or yes) were also evaluated. Nutritional status was obtained by the body mass index (BMI), which was calculated by the ratio of body mass, in kilograms, to height, in meters, squared (BMI = kg/m^2^ = body mass/height^2^). Weight and height were measured using a high-precision scale coupled to the BOD POD and an Altura Exata stadiometer, respectively (Belo Horizonte, Brazil). Nutritional status was classified based on BMI by age, according to the reference values of the growth curves of the World Health Organization (WHO) [53], categorized as low weight or adequate BMI (male, BMI ≤ 24.9 kg/m^2^ at 18 years old or BMI ≤ 25.4 kg/m^2^ at 19 years old; female, BMI ≤ 24.8 kg/m^2^ at 18 years old or BMI ≤ 25.0 kg/m^2^ at 19 years old), and overweight or obesity (male, BMI > 24.9 kg/m^2^ at 18 years old or BMI > 25.4 kg/m^2^ at 19 years old; female, BMI > 24.8 kg/m^2^ at 18 years old or BMI > 25.0 kg/m^2^ at 19 years old).

### 2.5. Statistical Analysis

Descriptive analyses were performed for (dis)satisfaction with body image according to exposure, and the prevalence and respective intervals were estimated with 95% confidence (95%CI). The chi-square test was used to examine the differences between the prevalence of body (dis)satisfaction categories and the characteristics of the sample. The level of statistical significance was set at *p* ≤ 0.05. The prevalence of body (dis)satisfaction categories for each food group according to the degree of processing was assessed using analysis of variance (ANOVA). The consumption of culinary preparations, processed, and ultra-processed foods related to (dis)satisfaction with body image was evaluated using multinomial logistic regression analysis (crude and adjusted models), calculating the Odds Ratio (OR) with their respective 95% confidence intervals (95%CI). The satisfaction with the body image category was considered a reference in the multinomial regression models. The models were adjusted for age, skin color, years of schooling, economic class, leisure time physical activity, screen time, alcohol consumption, smoking, and BMI. The analyses were performed by using the statistical program Stata version 14.

### 2.6. Ethics Statements

The study was approved by the Research Ethics Committee of the University Hospital of the Federal University of Maranhão (consubstantiated opinion No. 1,302,489 of 29 October 2015). Written informed consent was obtained from all subjects by signing the informed consent form.

## 3. Results

### 3.1. Population Characteristics

Of the 2515 study participants, 52.5% were female, 69.3% were 18 years old, 63.6% had brown skin color, 88.0% had 9 to 11 years of schooling, 50.1% were from economic class C, and 70.1% had a family income ≤3 minimum wages. Regarding lifestyle, 53.0% did not practice leisure time physical activity, 63.4% had more than 5 h of screen time per day, 41.5% consumed alcoholic beverages, and 3.5% were smokers (Table 1).

### 3.2. Body Image (Dis)satisfaction

In the sample, 77% of adolescents considered themselves dissatisfied with their body image, with 42.8% dissatisfied with thinness and 34.2% dissatisfied with excess weight. The differences by gender were statistically significant (*p* < 0.001), with the prevalence of dissatisfaction with thinness being higher among boys (54.7%) than among girls (32.7%), and dissatisfaction with excess weight being more prevalent among girls (44.8%) than among boys (22.9%). 

### 3.3. Food Intake and Body Image (Dis)satisfaction

Food consumption according to each group of the NOVA classification was evaluated according to (dis)satisfaction with body image, demonstrating greater consumption of culinary preparations, such as fruits, chicken, fish, and seafood (*p* < 0.001), among those dissatisfied with excess weight and higher consumption of rice (*p* < 0.001), processed foods (*p* < 0.001), and industrialized bread (*p* < 0.001) among adolescents dissatisfied with thinness (Table 2).

### 3.4. Association between Food Consumption According to the Degree of Processing and Body Image (Dis)satisfaction

Table 3 shows the association between food consumption according to the degree of processing in tertiles and (dis)satisfaction with body image. In the crude analysis, the third tertile of consumption of processed foods was associated with dissatisfaction with excess weight. However, in the adjusted analysis, the association was not maintained. Regarding the consumption of culinary preparations and ultra-processed foods, there was no association with body (dis)satisfaction.

## 4. Discussion

The results showed a high prevalence of body image dissatisfaction among adolescents in the city of São Luís, Brazil. However, food consumption according to the degree of processing was not associated with body (dis)satisfaction.

Regarding the high prevalence of dissatisfaction with body image (77%), the findings corroborate other studies conducted in Brazil with adolescents aged between 10 and 16 years [35,54,55]. For example, Andrade, et al., [35] reported 74% of adolescent body dissatisfaction. In Pelotas [55], body dissatisfaction reached 51.0% of boys and 65.6% of girls. Several aspects may influence body satisfaction in this age group, such as changes in appearance and body shape typical of puberty [2], which go against the social and cultural pressures of worshiping the “beautiful body” [56]. These factors lead to insecurity and excessive concern with weight and body image at this age [57]. 

This study showed a higher percentage of dissatisfaction with thinness (42.8%) than with excess weight (34.2%), corroborating findings among adolescents aged between 12 and 18 years in Tunisia [32], with a higher percentage of dissatisfaction with thinness (27%) than the percentage of dissatisfaction due to excess weight (15.4%). However, these results differ from those of other studies [55,58], which identified more dissatisfaction with excess weight than with thinness among adolescents. Among adolescents’ main reasons for body dissatisfaction, aesthetics, self-esteem, and health stand out [59]. From an aesthetic perspective, there is a tendency to idealize unreal beauty standards proposed by the media and social and cultural environments [32]; the ideal of thinness for females [60], and the muscular standard for males [61]. This tendency may explain the differences in the body (dis)satisfaction by gender in this sample, which have been previously studied by Soares Filho, et al., [62], demonstrating a greater chance of dissatisfaction with body image due to the desire to lose weight in females when compared with males.

In this study, rice and industrialized bread consumption were higher among adolescents dissatisfied with thinness. Similarly, a study [13] conducted with Brazilian adolescents aged 12 to 17 identified that those who believed they were underweight consumed more bread. The high consumption of high-energy foods among adolescents can be justified by the desire to gain weight [30]. However, this theory assumes body image as exposure and food consumption as the outcome, which is the reverse causality of the hypothesis proposed in this study. Adolescents dissatisfied with excess weight showed higher consumption of fruits, poultry, and fish. This demonstrates a trend towards healthier food consumption among young people who want to lose weight. However, one study conducted with Brazilian students aged 15 to 20 years [63] and another with Mauritian students aged 14 to 17 years [17] found different results, in which the consumption of fruits and vegetables was more frequent in adolescents satisfied with their body image.

Regarding the consumption of culinary preparations and processed food groups, no significant association with any category of body (dis)satisfaction was observed. However, Hernández Camacho, et al., [64], who investigated self-perception of weight (outcome) in a school sample from Spain, found divergent results since they reported an association between positive self-perception of weight and vegetable consumption. To date, we have found no other studies that have investigated the association between groups of culinary preparations and processed foods with body (dis)satisfaction in adolescents using the causality proposed in this study. Therefore, further research is suggested to investigate this relationship.

Adolescence is marked by greater consumption of ultra-processed foods [65], which, due to being high in calories, tend to increase the prevalence of obesity [66] and body fat levels [67], which can lower satisfaction with body image [5]. In addition, ultra-processed foods are highly palatable and rich in sugars and fats, which can trigger binge eating and contribute to body dissatisfaction [68]. However, in this study, the consumption of ultra-processed foods was not associated with any category of body (dis)satisfaction. Corroborating these findings, Andrade, et al., [35], in a study conducted with Brazilian students aged 10 to 14 years, also did not establish an association between the consumption of ultra-processed foods and (dis)satisfaction with body image. Santana, et al., [5] investigated the relationship between diet sodas and sugary drinks—foods from the ultra-processed group—with body dissatisfaction among adolescents aged between 10 and 17 years in Salvador, Brazil, and found no association. 

Note that body image can be represented by two different dimensions: perceptual and attitudinal. The perceptual dimension encompasses the individual’s perception of body size and shape. The attitudinal dimension comprises the subject’s emotions and attitudes towards their body [3,69,70]. Thus, the body’s self-perception corresponds to the perceptual component of the body image. At the same time, the feelings of satisfaction or dissatisfaction resulting from this perception inform the attitudinal component. Evidence shows that the attitudinal component of body image is more influenced by food intake than the perceptual component [24], which appears to be more stable [69]. However, body (dis)satisfaction was also a stable component in this study, considering that it was not influenced by food intake.

Few studies have investigated the association of food consumption according to the degree of food processing with body (dis)satisfaction in adolescents [35] and young adults [23], as mentioned above. Additionally, among those who investigated this association, most did not use the NOVA classification [5,11,30,32], included other age groups [14,24,33], and used body image as an independent variable [11,14,24,32]. In contrast, this study used body image as a dependent variable, considering evidence that worse diet profiles can lead to increased rates of excess weight [65] and changes in body self-perception [64]. However, it is essential to consider the possible bidirectional association between food consumption and body dissatisfaction, especially in the long term. Therefore, it is suggested that new studies, especially longitudinal studies, be carried out to elucidate the cause-and-effect relationship between food consumption and body image.

The main strengths of this study are the large sample size, the use of validated instruments, the use of highly valid methods for measuring body composition, and the use of the NOVA food classification. Additionally, the silhouette scale allows the analysis of issues that require the individual’s opinion since it is a subjective measure [55].

One limitation of this study was the sample loss due to difficulties locating the adolescents, which required incorporating other individuals not in the original cohort. Additionally, memory bias in response to food consumption questions and overestimation of consumption by the FFQ can also be considered limitations of this study. These limitations occur because the same food can fit into more than one group due to the difficulty of fitting it into a single category according to the degree of processing. To minimize these limitations, the interviewers were trained to minimize recall bias, ensuring proper questionnaire application. Furthermore, to minimize the overestimation of consumption caused by the difficulty of separating foods between groups, each food was assigned the NOVA classification category in which it is most used (information from the 2008–2009 Family Budget Survey [44] for the state of Maranhão were used in these cases). Moreover, the difficulty of inferring causality due to the study’s cross-sectional design and the scarcity of studies that allow data comparison were also limitations since few used the classification of foods by the degree of processing and the same causality proposed in this study.

Despite the lack of association between the consumption of culinary preparations, processed or ultra-processed foods, and body (dis)satisfaction in adolescents, most of the young people in this study were dissatisfied with their body image, mainly with thinness. Note the seriousness of these results since body dissatisfaction is linked to several determinants related to health, which can lead to anxiety [71], depression, and low self-esteem [7], in addition to being a trigger for risk behaviors for food disorders [72]. Considering that adolescence is a period of transition that may influence habits in adult life, the results of this study are relevant to encourage a change in practices at this stage of life, highlighting the need for measures to reduce rates of body dissatisfaction among adolescents.

## 5. Conclusions

This study provided relevant information about the high prevalence of body image dissatisfaction among adolescents but showed no association between body (dis)satisfaction and food consumption according to the degree of food processing.

## Figures and Tables

**Table 1:** Characterization of the general sample and prevalence of (dis)satisfaction with body image according to sociodemographic, economic, anthropometric, and behavioral characteristics in adolescents. São Luís, Maranhão, Brazil, 2016/2017.

	General	(Dis)satisfaction with Body Image			
	*n* (%)	Satisfied *n* (%)	Dissatisfaction with Thinness *n* (%)	Dissatisfaction with Excess Weight *n* (%)	*p*-Value
Sociodemographic and economic					
Gender (*n* = 2515)					<0.001
Male	1196 (47.5)	266 (22.4)	648 (54.7)	271 (22.9)	
Female	1319 (52.5)	295 (22.5)	429 (32.7)	589 (44.8)	
Age (*n* = 2515)					0.034
18 years	1742 (69.3)	394 (22.8)	766 (44.4)	566 (32.8)	
19 years	773 (30.7)	167 (21.6)	311 (40.3)	294 (38.1)	
Skin Color (*n* = 2500)					0.046
White	495 (19.8)	111 (22.6)	187 (38.1)	193 (39.3)	
Black	416 (16.6)	96 (23.2)	172 (41.5)	146 (35.3)	
Brown	1589 (63.6)	350 (22.2)	713 (45.1)	516 (32.7)	
Years of schooling (*n* = 2500)					0.374
0 to 8 years	136 (5.4)	32 (24.2)	59 (44.7)	41 (31.1)	
9 to 11 years	2199 (88.0)	493 (22.5)	951 (43.5)	745 (34.0)	
12 years or more	165 (6.6)	34 (20.6)	63 (38.2)	68 (41.2)	
Economic class (*n* = 2226)					0.011
A–B	660 (29.7)	155 (23.6)	240 (36.6)	261 (39.8)	
C	1116 (50.1)	251 (22.6)	496 (44.7)	362 (32.6)	
D–E	450 (20.2)	98 (22.0)	193 (43.3)	155 (34.7)	
Family income (*n* = 2238)					0.264
≤3 MW	1569 (70.1)	337 (21.6)	693 (44.5)	528 (33.9)	
3.1 to 6 MW	441 (19.7)	100 (22.8)	184 (42.0)	154 (35.2)	
>6 MW	228 (10.2)	49 (21.7)	85 (37.6)	92 (40.7)	
Lifestyle					
Leisure Time Physical Activity (*n* = 2515)					<0.001
Yes	1183 (47.0)	256 (21.7)	464 (39.3)	461 (39.0)	
No	1332 (53.0)	305 (23.2)	613 (46.5)	399 (30.3)	
Screen time (*n* = 2269)					0.347
No use or use ≤ 2 h	245 (10.8)	58 (23.8)	118 (48.3)	68 (27.9)	
>2 to ≤5 h	586 (25.8)	131 (22.4)	252 (43.1)	202 (34.5)	
>5 h	1438 (63.4)	323 (22.5)	617 (43.0)	496 (34.5)	
Consumption of alcoholic beverages (*n* = 2494)					0.921
No	1459 (58.5)	323 (22.2)	626 (43.0)	507 (34.8)	
Yes	1035 (41.5)	235 (22.7)	446 (43.1)	353 (34.2)	
Smoking (*n* = 2515)					0.810
No	2426 (96.5)	541 (22.5)	1036 (43.0)	832 (34.5)	
Yes	89 (3.5)	20 (22.5)	41 (46.1)	28 (31.4)	
Anthropometric variables and indicators					
BMI (*n* = 2515)					<0.001
Adequate/Low weight	1998 (79.4)	500 (25.2)	1059 (53.3)	427 (21.5)	
Overweight/obesity	517 (20.6)	61 (11.9)	18 (3.5)	433 (84.6)	

MW: Minimum wage; BMI: Body mass index. *p*-values calculated by chi-square test.

**Table 2:** Food intake according to the prevalence of body image (dis)satisfaction in adolescents. São Luís, Maranhão, Brazil. 2016/2017.

Diet	Satisfied Mean Energy Intake	Dissatisfied (Thinness) Mean Energy Intake	Dissatisfied (Excess Weight) Mean Energy Intake	*p*-Value
**%kcal culinary preparations**	57.5	56.7	58.0	0.09
Rice	15.8	17.7	15.1	<0.001
Beans	3.7	3.8	3.6	0.26
Milk	5.6	5.2	5.5	0.29
Other Cereals	8.3	8.1	8.2	0.79
Vegetables	3.4	3.2	3.3	0.48
Fruits	6.9	6.1	7.3	<0.001
Red Meats	4.2	3.9	4.0	0.16
Chicken/Poultry	2.8	2.8	3.2	<0.001
Fish/Seafood	2.4	2.2	3.0	<0.001
Eggs	1.6	1.5	1.7	0.11
Coffee	0.6	0.6	0.7	0.77
Chestnuts	0.4	0.4	0.4	0.42
**%kcal processed**	4.5	4.7	4.0	<0.001
**%kcal ultra-processed**	34.7	35.5	34.5	0.28
Industrialized bread	3.9	4.3	3.4	<0.001
Cakes and biscuits	7.7	8.2	7.6	0.16
Goodies and sweets	5.4	5.5	5.9	0.07
Fast food	5.0	4.9	4.7	0.64
Sugary drinks	3.9	3.8	3.8	0.72
Salted biscuits and French fries	3.4	3.8	3.7	0.21
Cream cheese and industrialized yogurt	1.4	1.2	1.5	0.01
Margarine	1.2	1.2	1.1	0.71
Sausages and instant noodles	1.1	1.1	1.1	0.51
Ketchup and mayonnaise	0.5	0.5	0.5	0.60
Breakfast cereals and cereal bars	0.5	0.4	0.5	0.10

Means compared with ANOVA—Analysis of variance. %kcal: percentage of total energy intake.

**Table 3:** Association of food consumption according to the degree of processing with body image in adolescents. São Luís, Maranhão, Brazil, 2016/2017.

Variables	Dissatisfaction with Thinness				Dissatisfaction with Excess Weight			
	Crude Analysis		Adjusted Analysis *		Crude Analysis		Adjusted Analysis *	
	OR (95%CI)	*p*	OR (95%CI)	*p*	OR (95%CI)	*p*	OR (95%CI)	*p*
Culinary preparations (%)		0.336		0.613		0.306		0.466
Tertile 1	1		1		1		1	
Tertile 2	0.98 (0.77; 1.26)		1.17 (0.88; 1.55)		1.07 (0.82; 1.40)		1.06 (0.77; 1.47)	
Tertile 3	0.88 (0.69; 1.14)		1.07 (0.81; 1.43)		1.15 (0.88; 1.49)		1.13 (0.82; 1.55)	
Processed (%)		0.779		0.975		<0.001		0.073
Tertile 1	1		1		1		1	
Tertile 2	0.98 (0.76; 1.27)		0.92 (0.69; 1.23)		0.78 (0.60; 1.01)		0.92 (0.67; 1.26)	
Tertile 3	1.03 (0.80; 1.33)		0.99 (0.74; 1.32)		0.61 (0.47; 0.80)		0.74 (0.53; 1.02)	
Ultra-processed (%)		0.248		0.863		0.353		0.463
Tertile 1	1		1		1		1	
Tertile 2	1.10 (0.86; 1.42)		1.02 (0.77; 1.36)		0.95 (0.73; 1.23)		0.96 (0.70; 1.32)	
Tertile 3	1.16 (0.90; 1.49)		0.98 (0.73; 1.30)		0.88 (0.68; 1.15)		0.89 (0.64; 1.22)	

OR: Odds Ratio; 95%CI: 95% confidence interval; %: Percentage of total energy. * Adjusted for the variables: age, skin color, years of schooling, socioeconomic class, leisure time physical activity, screen time, alcohol consumption, smoking, and BMI. Obtained from multinomial regression.

## Data Availability

Restrictions apply to the availability of these data. Data was obtained from rosangela.flb@ufma.br and are available from the authors upon reasonable request and with the permission of Rosangela Fernandes Lucena Batista. The data presented in this study are not publicly available due to being used under license for the current study.

## Abbreviations

PeNSE	National Survey of School Health
BMI	Body Mass Index
RBI	Real Body Image
IBI	Ideal Body Image
FFQ	Food frequency questionnaire
TEV	Total Energy Value
ABEP	Brazilian Association of Research Companies
SAPAC	Self-Administered Physical Activity Checklist
AUDIT	Alcohol Use Disorder Identification Test
WHO	World Health Organization
OR	Odds Ratio
CI	Confidence interval

## References

[1] World Health Organization (1986). Young People’s Health—A Challenge for Society: Report of a WHO Study Group on Young People and Health for All by the Year 2000.

[2] Williams J.M., Currie C. (2000). Self-Esteem and Physical Development in Early Adolescence: Pubertal Timing and Body Image. J. Early Adolesc..

[3] Tavares M.d.C.G.C.F., Campana A.N.N.B., Tavares Filho R.F., Campana M.B. (2010). Avaliação perceptiva da imagem corporal: História, reconceituação e perspectivas para o Brasil. Psicol. Estud..

[4] Instituto Brasileiro de Geografia e Estatística (2016). Pesquisa Nacional de Saúde Escolar 2015.

[5] Santana M.L.P.D., Silva R.D.C.R., Assis A.M.O., Raich R.M., Machado M.E.P.C., Pinto E.D.J., de Moraes L.T.L.P., Ribeiro H.D.C. (2013). Factors associated with body image dissatisfaction among adolescents in public schools students in Salvador, Brazil. Nutr. Hosp..

[6] Markey C.N. (2010). Invited Commentary: Why Body Image is Important to Adolescent Development. J. Youth Adolesc..

[7] Xu X., Mellor D., Kiehne M., Ricciardelli L.A., McCabe M.P., Xu Y. (2010). Body dissatisfaction, engagement in body change behaviors and sociocultural influences on body image among Chinese adolescents. Body Image.

[8] Frank R., Claumann G.S., Felden É.P.G., Silva D.A.S., Pelegrini A. (2018). Body weight perception and body weight control behaviors in adolescents. J. Pediatr..

[9] Paxton S.J., Eisenberg M.E., Neumark-Sztainer D. (2006). Prospective predictors of body dissatisfaction in adolescent girls and boys: A five-year longitudinal study. Dev. Psychol..

[10] Bibiloni M.D.M., Pich J., Pons A., Tur J.A. (2013). Body image and eating patterns among adolescents. BMC Public Health.

[11] Zarychta K., Chan C.K.Y., Kruk M., Luszczynska A. (2020). Body satisfaction and body weight in under- and healthy-weight adolescents: Mediating effects of restrictive dieting, healthy and unhealthy food intake. Eat. Weight Disord..

[12] Deforche B., van Dyck D., Deliens T., de Bourdeaudhuij I. (2015). Changes in weight, physical activity, sedentary behaviour and dietary intake during the transition to higher education: A prospective study. Int. J. Behav. Nutr. Phys. Act..

[13] Madalosso M.M., Schaan B., Cureau F.V. (2020). Association between body weight perception and quality of diet in Brazilian adolescents. Rev. Paul. Pediatr..

[14] Araújo A.C., Oliveira A. (2018). (In)Satisfação com a imagem corporal: Associação com o consumo alimentar e a ingestão nutricional. Acta Port. Nutr..

[15] Burrows A., Cooper M. (2002). Possible risk factors in the development of eating disorders in overweight pre-adolescent girls. Int. J. Obes..

[16] Bearman S.K., Presnell K., Martinez E., Stice E. (2006). The skinny on body dissatisfaction: A longitudinal study of adolescent girls and boys. J. Youth Adolesc..

[17] Balluck G., Toorabally B.Z., Hosenally M. (2016). Association between body image dissatisfaction and body mass index, eating habits and weight control practices among mauritian adolescents. Malays. J. Nutr..

[18] Monteiro C.A. (2009). Nutrition and health. The issue is not food, nor nutrients, so much as processing. Public Health Nutr..

[19] Monteiro C.A., Cannon G., Levy R., Moubarac J.C., Jaime P., Martins A.P., Canella D., Louzada M., Parra D., Ricardo C. (2016). NOVA. A estrela brilha. World Nutr..

[20] Monteiro C.A., Levy R.B., Claro R.M., de Castro I.R.R., Cannon G. (2010). A new classification of foods based on the extent and purpose of their processing. Cad. Saude Publica.

[21] Monteiro C. (2010). The big issue is ultra-processing [Commentary]. World Nutr..

[22] Brasil (2014). Guia Alimentar Para a População Brasileira.

[23] Da Cunha P.R.F., Machado L.M.M. (2019). Avaliação do estado nutricional, satisfação com a imagem corporal, consumo e comportamento alimentar de discentes de balé clássico em uma escola de dança em Belém-PA. Rev. Bras. Obes. Nutr. Emagr..

[24] Oliveira N., Coelho G.M.D.O., Cabral M.C., Bezerra F.F., Faerstein E., Canella D.S. (2020). Association of body image (dis)satisfaction and perception with food consumption according to the NOVA classification: Pró-Saúde Study. Appetite.

[25] World Health Organization (2003). Diet, Nutrition and the Prevention of Chronic Diseases.

[26] Miles L. (2008). The new WCRF/AICR report—Food, nutrition, physical activity and the prevention of cancer: A global perspective. Nutr. Bull..

[27] Louzada M.L.D.C., Baraldi L.G., Steele E.M., Martins A.P.B., Canella D.S., Moubarac J.C., Levy R.B., Cannon G., Afshin A., Imamura F. (2015). Consumption of ultra-processed foods and obesity in Brazilian adolescents and adults. Prev. Med..

[28] Mendonça R.D.D., Pimenta A.M., Gea A., de La Fuente-Arrillaga C., Martinez-Gonzalez M.A., Lopes A.C.S., Bes-Rastrollo M. (2016). Ultraprocessed food consumption and risk of overweight and obesity: The University of Navarra Follow-Up (SUN) cohort study. Am. J. Clin. Nutr..

[29] Derenne J.L., Beresin E.V. (2006). Body image, media, and eating disorders. Acad. Psychiatry.

[30] Oellingrath I.M., Hestetun I., Svendsen M.V. (2016). Gender-specific association of weight perception and appearance satisfaction with slimming attempts and eating patterns in a sample of young Norwegian adolescents. Public Health Nutr..

[31] Chen H.Y., Lemon S.C., Pagoto S.L., Barton B.A., Lapane K.L., Goldberg R.J. (2014). Personal and parental weight misperception and self-reported attempted weight loss in US children and adolescents, National Health and Nutrition Examination Survey, 2007–2008 and 2009–2010. Prev. Chronic Dis..

[32] Ben Ayed H., Yaich S., ben Jemaa M., ben Hmida M., Trigui M., Jedidi J., Sboui I., Karray R., Feki H., Mejdoub Y. (2019). What are the correlates of body image distortion and dissatisfaction among school-adolescents. Int. J. Adolesc. Med. Health.

[33] Resende A.D.S., Santos L.R., Leite M.D.M.R., Raposo O.F.F., Netto R.S.M. (2019). Eating habits and body image among gym goers. Mundo Saude.

[34] Ribeiro-Silva R.D.C., Fiaccone R.L., Conceição-Machado M.E.P.D., Ruiz A.S., Barreto M.L., Santana M.L.P. (2018). Body image dissatisfaction and dietary patterns according to nutritional status in adolescents. J. Pediatr..

[35] Andrade L.M.M., Costa J.A., Carrara C.F., Netto M.P., Cândido A.P.C., Oliveira e Silva R.M.S., Mendes L.L. (2019). Estado nutricional, consumo de alimentos ultraprocessados e imagem corporal de adolescentes de uma escola privada do município de Juiz de Fora—MG. HU Rev..

[36] Confortin S.C., Ribeiro M.R.C., Barros A.J.D., Menezes A.M.B., Horta B.L., Victora C.G., Barros F.C., Gonçalves H., Bettiol H., dos Santos I.S. (2021). RPS Brazilian Birth Cohorts Consortium (Ribeirão Preto, Pelotas and São Luís): History, objectives and methods. Cad. Saude Publica.

[37] Simões V.M.F., Batista R.F.L., Alves M.T.S.S.D.B.E., Ribeiro C.C.C., Thomaz E.B.A.F., Carvalho C.A.D., Silva A.A.M. (2020). Saúde dos adolescentes da coorte de nascimentos de São Luís, Maranhão, Brazil, 1997/1998. Cad. Saude Publica.

[38] Stunkard A.J., Sørensen T., Schulsinger F. (1983). Use of the Danish Adoption Register for the study of obesity and thinness. Res. Publ. Assoc. Res. Nerv. Ment. Dis..

[39] Felden É.P.G., Claumann G.S., Sacomori C., Daronco L.S.E., Cardoso F.L., Pelegrini A. (2015). Fatores sociodemográficos e imagem corporal em adolescentes do ensino médio. Ciênc. Saude Colet..

[40] Schneider B.C., Motta J.V.D.S., Muniz L.C., Bielemann R.M., Madruga S.W., Orlandi S.P., Gigante D.P., Assunção M.C.F. (2016). Desenho de um questionário de frequência alimentar digital autoaplicado para avaliar o consumo alimentar de adolescentes e adultos jovens: Coortes de nascimentos de Pelotas, Rio Grande do Sul. Rev. Bras. Epidemiol..

[41] Bogea E.G., França A.K.T.C., Bragança M.L.B.M., Vaz J.S., Assunção M.C., Barbieri M.A., Bettiol H., Silva A.A.M. (2021). Relative validity of a food frequency questionnaire for adolescents from a capital in the Northeastern region of Brazil. Braz. J. Med. Biol. Res..

[42] Benzecry E.H., Pinheiro A.B.V., Lacerda E.M.A., Gomes M.C.S., Costa V.M. (2005). Tabela Para Avaliação de Consumo Alimentar em Medidas Caseiras.

[43] Taco (2011). Tabela Brasileira de Composição de Alimentos.

[44] Instituto Brasileiro de Geografia e Estatística (2011). Pesquisa de Orçamentos Familiares 2008–2009: Tabelas de Composição Nutricional dos Alimentos Consumidos no Brasil.

[45] Pinheiro A.B.V., Lacerda E.M.D.A., Benzecry E.H., Gomes M.C.D.S., da Costa V.M. (2002). Tabela Para Avaliação de Consumo Alimentar em Medidas Caseiras.

[46] Monteiro C.A., Moubarac J.C., Levy R.B., Canella D.S., Louzada M.L.D.C., Cannon G. (2018). Household availability of ultra-processed foods and obesity in nineteen European countries. Public Health Nutr..

[47] ABEP Critério de Classificação Econômica Brasil—CCEB 2018. https://www.abep.org/criterio-brasil.

[48] Sallis J.F., Strikmiller P.K., Harsha D.W., Feldman H.A., Ehlinger S., Stone E.J., Williston J., Woods S. (1996). Validation of interviewer- and self-administered physical activity checklists for fifth grade students. Med. Sci. Sports Exerc..

[49] Ainsworth B.E., Haskell W.L., Leon A.S., Jacobs D.R., Montoye H., Sallis J.F., Paffenbarger R.S. (1993). Compendium of physical activities: Classification of energy costs of human physical activities. Med. Sci. Sports Exerc..

[50] World Health Organization Global Recommendations on Physical Activity for Health. https://www.who.int/dietphysicalactivity/factsheet_recommendations/en/.

[51] Garmy P., Ward T.M. (2017). Sleep Habits and Nighttime Texting among Adolescents. J. Sch. Nurs..

[52] Moretti-Pires R.O., Corradi-Webster C.M. (2011). Adaptação e validação do Alcohol Use Disorder Identification Test (AUDIT) para população ribeirinha do interior da Amazônia, Brasil. Cad. Saude Publica.

[53] de Onis M., Onyango A.W., Borghi E., Siyam A., Nishida C., Siekmann J. (2007). Development of a WHO growth reference for school-aged children and adolescents. Bull. World Health Organ..

[54] Bilhar K.P., Marcadenti A., Conde S.R. (2016). Estado nutricional, consumo de macronutrientes e (in)satisfação corporal em atletas adolescentes de voleibol. Rev. Bras. Nutr. Esport..

[55] Dumith S.D.C., Menezes A.M.B., Bielemann R.M., Petresco S., da Silva I.C.M., Linhares R.D.S., Amorim T.C., Duarte D.V., Araújo C.L.P., dos Santos J.V. (2012). Insatisfação corporal em adolescentes: Um estudo de base populacional. Ciênc. Saude Colet..

[56] Alves D., Pinto M., Alves S., Mota A., Leirós V. (2009). Cultura e imagem corporal. Motricidade.

[57] Pereira A.M.G.R. (2016). Preocupação com o peso e prática de dietas por adolescentes. Acta Port. Nutr..

[58] Marques M.I., Pimenta J., Reis S., Ferreira L.M., Peralta L. (2016). (In) Satisfação com a imagem corporal na adolescência. Nascer Crescer.

[59] Petroski E.L., Pelegrini A., Glaner M.F. (2012). Reasons and prevalence of body image dissatisfaction in adolescents. Ciênc. Saude Colet..

[60] Stice E., Whitenton K. (2002). Risk factors for body dissatisfaction in adolescent girls: A longitudinal investigation. Dev. Psychol..

[61] Nye S. (2007). The Muscular Ideal: Psychological, Social and Medical Perspectives.

[62] Soares Filho L.C., Batista R.F.L., Cardoso V.C., Simões V.M.F., Santos A.M., Coelho S.J.D.D.A.C., Silva A.A.M. (2020). Body image dissatisfaction and symptoms of depression disorder in adolescents. Braz. J. Med. Biol. Res..

[63] Santos E.M.C., Tassitano R.M., do Nascimento W.M.F., Petribú M.D.M.V., Cabral P.C. (2011). Satisfação com o peso corporal e fatores associados em estudantes do ensino médio. Rev. Paul. Pediatr..

[64] Camacho J.D.H., Lazo M.R., Ríos P.B., Prieto I.R., Jáuregui-Lobera I. (2015). Hábitos alimentarios, sobrecarga ponderal y autopercepción del peso en el ámbito escolar. Nutr. Hosp..

[65] Taylor A.L., Jacobson M.F. (2016). Carbonating the World: The Marketing and Health Impact of Sugar Drinks in Low- and Middle-Income Countries.

[66] Mistry S.K., Puthussery S. (2015). Risk factors of overweight and obesity in childhood and adolescence in South Asian countries: A systematic review of the evidence. Public Health.

[67] Costa C.S., Del-Ponte B., Assunção M.C.F., Santos I.S. (2018). Consumption of ultra-processed foods and body fat during childhood and adolescence: A systematic review. Public Health Nutr..

[68] Wu Y.K., Zimmer C., Munn-Chernoff M.A., Baker J.H. (2020). Association between food addiction and body dissatisfaction among college students: The mediating role of eating expectancies. Eat. Behav..

[69] Vocks S., Legenbauer T., Heil A. (2007). Food intake affects state body image: Impact of restrained eating patterns and concerns about eating, weight and shape. Appetite.

[70] Hayes J.F., D’Anci K.E., Kanarek R.B. (2011). Foods that are perceived as healthy or unhealthy differentially alter young women’s state body image. Appetite.

[71] Presnell K., Stice E., Seidel A., Madeley M.C. (2009). Depression and eating pathology: Prospective reciprocal relations in adolescents. Clin. Psychol. Psychother..

[72] Fortes L.D.S., Filgueiras J.F., Oliveira F.D.C., Almeida S.S., Ferreira M.E.C. (2016). Modelo etiológico dos comportamentos de risco para os transtornos alimentares em adolescentes brasileiros do sexo feminino. Cad. Saude Publica.

